# Comparison of the effect of calcium gluconate and batroxobin on the release of transforming growth factor beta 1 in canine platelet concentrates

**DOI:** 10.1186/1746-6148-8-121

**Published:** 2012-07-25

**Authors:** Raul F Silva, Jorge U Carmona, Cleuza MF Rezende

**Affiliations:** 1 Departamento de Clínica e Cirurgia Animal, Universidade Federal de Minas Gerais, Belo Horizonte, Brazil; 2 Grupo de Investigación Terapia Regenerativa, Departamento de Salud Animal, Universidad de Caldas, Manizales, Colombia

**Keywords:** Tube method, Platelet Rich-Plasma, Dog, Regenerative medicine

## Abstract

**Background:**

The clinical use of autologous platelet concentrates (also known as platelet-rich plasma) on the field of regenerative therapy, in the last decade has been the subject of several studies especially in equine medicine and surgery. The objectives of this study was: 1) to describe and compare the cellular population in whole blood, lower fraction (A) and upper fraction (B) of platelet concentrates, 2) to measure and compare the transforming growth factor beta 1 (TGF-β_1_) concentration in plasma and both platelet concentrates after be activated with calcium gluconate or batroxobin plus calcium gluconate and, 3) to determine correlations between cell counts in platelet concentrates and concentrations of TGF-β_1_. Blood samples were taken from 16 dogs for complete blood count, plasma collection and platelet concentrates preparation. The platelet concentrates (PC) were arbitrarily divided into two fractions, specifically, PC-A (lower fraction) and PC-B (upper fraction). The Platelet concentrates were analyzed by hemogram. After activated with calcium gluconate or batroxobin plus calcium gluconate, TGF-β_1_ concentration was determined in supernatants of platelet concentrates and plasma.

**Results:**

There were differences statistically significant (P < 0.05) for the platelet count and leukocyte count and TGF-β_1_ concentration between whole blood, plasma and both platelet concentrates. A significant correlation was found between the number of platelets in both platelet concentrates and TGF-β_1_ concentration. Platelet collection efficiency was 46.34% and 28.16% for PC-A and PC-B, respectively. TGF-β_1_ concentration efficiency for PC activated with calcium gluconate was 47.75% and 31.77%, for PC-A and PC-B, respectively. PC activated with batroxobin plus CG showed 46.87% and 32.24% for PC-A and PC-B, respectively.

**Conclusions:**

The methodology used in this study allows the concentration of a number of platelets and TGF-β_1_ that might be acceptable for a biological effect for clinical or experimental use as a regenerative therapy in dogs.

## Background

The healing process is directed by complex biological mechanisms that involve many cells and proteins, such as cytokines and growth factors (GF), among others. Cellular and molecular interactions allow, under physiological conditions, the repair or regeneration of damaged tissues [1,2]. Platelets play a central role in the healing process. These cytoplasmic fragments not only have hemostatic properties [3], but also have pro-inflammatory, regulatory [4] and regenerative properties, which are mediated by interaction with other cells (neutrophils and endothelial cells), GF, chemokines and other regulatory molecules [5].

Currently, there is a growing interest in the use of autologous platelet concentrates (PC), also known as platelet rich plasma (PRP) for stimulating the healing process and promoting regeneration instead of reparation. Platelet alpha granules [4,6] contain at least seven GF directly involved in the healing process. Of these proteins, transforming growth factor beta isoform 1 (TGF-β_1_) is of pivotal importance for its actions on cell proliferation, angiogenesis and extracellular matrix deposition [7].

Many substances could potentially be used for platelet activation as a previous step for the clinical use PC. Platelet activation understood as a combination of fibrinogen cleavage leading to fibrin mesh formation and externalization of alpha granules containing growth factors [8]. Arbitrarily, activating substances could are classified according to their chemical structure in proteic, non-proteic forms and combination of both. Proteic activating substances include thrombin (either from bovine or autogenous sources) [9,10], and soluble collagen type I [11] amongst others. Calcium salts, such as calcium chloride and calcium gluconate [9] are classified as non-proteic activating substances. Combination of both includes batroxobin plus calcium gluconate [10].

Some protocols for platelet activation include the use of a calcium salt solely and others include the combination of a calcium salt plus a proteic activating substance. To date, there is no convincing data about what is the best activating substance (in terms of GF concentration) for PC activation and more research is necessary to know the effect of these substances (either alone or combined) on the release of platelet main derived GF, such as TGF-β_1_.

There is information on basic biological aspects and clinical use of PC in musculoskeletal and soft tissue injuries in human beings [12-15] and horses [16-18]. The study presented here provides the description of a manual protocol for producing two kinds of canine PC arbitrarily classified as lower fraction (PC-A) and upper fraction (PC-B) and evaluates the effect of a non-proteic activating substance (calcium gluconate -CG-) and the combination of a proteic activating substance (batroxobin) plus CG on the release of TGF-β_1_ from canine PC.

The aims of this study were: 1) to describe and compare the cellular population in whole blood, lower fraction (A) and upper fraction (B) of platelet concentrates (PC), 2) to measure and compare the of TGF-β_1_ concentration plasma in both PC after be activated with calcium gluconate (CG) or batroxobin plus CG and, 3) to determine correlations between cell counts in PC and concentrations of TGF-β_1_.

## Results

### Cells

Platelet, WBC, and GRA counts were significantly different (P < 0.01) between whole blood and both PC. PC-A showed the highest PLT and WBC counts. GRA concentration was highest in whole blood. The absolute count of LYM was significantly lower (P < 0.01) in whole blood and PC-B in comparison with PC-A. However, the relative count of LYM was significantly lower (P < 0.01) in whole blood in comparison with both PC. Absolute and relative counts of MID were similar for each blood component. MPV was significantly higher (P < 0.01) in PC-A, in comparison with whole blood and PC-B. PDW was significantly different between each blood component (Table 1).

**Table 1:** **General results of the cellular variables.** Data presented as mean (standard error)

**Variable**	**Blood component**		
	**Whole Blood**	**Platelet Concentrate-A**	**Platelet Concentrate-B**
PLT × 10^3^/μL	330.75 (18.28) a	1072.90 (84.01)b	652.00 (56.47)c
PCV %	45.50 (1.05) a	5.81 (1.01) a	1.58 (0.84) c
WBC × 10^3^/μL	10.94 (0.77) a	17.86 (2.56) b	4.46 (1.27) c
LYM × 10^3^/μL	2.27 (0.25) a	11.38 (1.63) b	3.44 (1.26) a
LYM %	20.97 (2.08) a	65.78 (3.41) b	67.18 (6.82) b
MID × 10^3^/μL	0.49 (007) a	0.61 (0.21) a	0.23 (0.10) a
MID %	4.49 (0.55) a	3.08 (0.79) a	3.60 (0.93) a
GRA × 10^3^/μL	8.20 (0.68) a	5.85 (1.11) b	0.77 (0.22) c
GRA %	74.54 (2.01) a	31.14 (3.60) b	29.22 (2.38) c
MPV (fL)	8.36 (0.18) a	8.80 (0.17) b	8.31 (0.13) a
PDW %	35.53 (0.47) a	36.79 (0.37) b	35.45 (0.45) a

Statistically significant differences by SNK test. ^a,b,c^ Different letters represent significant (P < 0.01).

### Total protein concentration

Total protein (mg/mL) concentration was similar for each blood component (Table 2).

**Table 2:** **Concentration of transforming growth factor beta 1 in plasma and supernatants of platelet concentrates (PC).** Data presented as mean (standard error)

**Activating substance**	**Variable**	**Blood component**		
		**Plasma**	**PC-A**	**PC-B**
Calcium Gluconate	TGF-β_1_ (ng/mL)	13.7 (2.88) a	45.8 (5.43) b	30.5 (3.11) c
	TP (mg/mL)	62.0 (0.96) a	62.3 (0.79) a	62.6 (1.06) a
	TGF-β_1_ (ng/mg of TP)	0.22 (0.05) a	0.73 (0.09) b	0.49 (0.05) c
Batroxobin plus calcium gluconate	TGF-β_1_ (ng/mL)	13.7 (2.88) a	44.9 (5.65) b	30.9 (3.20) c
	TP (mg/mL)	62.0 (0.96) a	63.1 (1.25) a	64.7 (1.65) a
	TGF-β_1_ (ng/mg of TP)	0.22 (0.05) a	0.72 (0.09) b	0.48 (0.05) c

Statistically significant differences by SNK test. ^a,b,c^ Different letters represent significant (P < 0.01).

### Transforming growth factor beta 1 concentration

Independently of the activating substance used, the TGF-β_1_ concentrations (ng/mL or ng/mg total protein) were significantly different (P < 0.01) between plasma and both PC. The highest TGF-β_1_ concentration was found in PC-A. No differences were noted between the two activating substances on the release of TGF-β_1_ of both PC (Table 2).

### Correlations

Highly significant correlations were found for PLT count and TGF-β_1_ concentration in both PC, independently of the activating substance used. For platelet concentrates activated with CG the correlation coefficients were ρ = 0.7 (P < 0.01) and ρ = 0.75 (P < 0.01) for PC-A and PC-B, respectively. For platelet concentrates activated with batroxobin plus CG the correlation coefficients were ρ = 0.71 (P < 0.01) and ρ = 0.75 (P < 0.01) for PC-A and PC-B, respectively.

### Collection (concentration) efficiency

Platelet collection efficiency was 46.34% and 28.16% for PC-A and PC-B, respectively. The combined collection efficiency for both PC was 74.5%. Platelet concentration was 224.38% for PC-A and 97.13% for PC-B in comparison with platelet counts in whole blood. Data of TGF-β_1_ concentration efficiency for each PC after activation with CG and batroxobin plus CG is presented in Table 3.

**Table 3:** Concentration efficiency of transforming growth factor beta 1 in supernatants of platelet concentrates (PC)

**Activating substance**	**Variable**	**Blood component**		
		**PC-A**	**PC -B**	**PC-A + B**
Calcium gluconate	TGF-β_1_ (%)	47.8	31.8	165.57
Batroxobin plus calcium gluconate	TGF-β_1_ (%)	46.9	32.2	158.22

## Discussion

This research describes a reliable method for producing platelet concentrates and consequently for concentrating GF, such as TGF-β_1_ from canine blood. Some manual (tube) protocols [19-22] and a semi-automated method [23] have been described for producing PC in dogs. Manual protocols were performed with either sodium citrate [19-21] or ACD-A [22] as anticoagulant. Those protocols included simple and double centrifugation steps for concentrating between 400 X 10^3^ to 1300 X 10^3^ PLT/μL. However, platelets were counted manually (light microscopy) and did not report additional hematologic features of the resulting PC.

Platelet concentration reached in the protocol described here was slightly lower than a semi-automated double centrifugation method evaluated in dogs, which presented a median concentration of 1336 PLT X 10^3^/μL [23]. However, this method is limited because it could only be used in medium at large breed dogs, since it requires 60 mL of blood for PC preparation. The protocol described here presents the advantage that PC is easily obtained by using one centrifugation step with a small volume of blood. This last situation is important when pediatric patients or small breed dogs are treated.

The size and weight of the blood cells, the relative centrifugation forces (g) and time are factors that determine the cellular and molecular characteristics of a PC. This concept is necessary for comparing the results of the research described here with other published studies in human beings [12-15] and horses [16-18]. The protocol described here permitted obtaining two kinds of different PC. Platelet concentrate-A presented a higher concentration of PLT/μL, WBC/μL and TGF-β_1_/mL (independently of the activating substance used) in comparison with PC-B. From a comparative point of view both canine and human blood present the same trend when centrifuged for PC preparation. However, equine blood requires a double centrifugation [24] for obtaining a PC with an acceptable quantity of PLT.

There are a lot of controversies about the ideal number of concentrated platelets in a PC for its clinical use in human beings and horses. Some researches consider the highest number of platelets concentrated in PRP to yield the best clinical results. This assumption could emerge from experimental results observed in rabbits where higher platelet concentrations were better for osteointegration, than lower platelet concentration [25]. However, excellent clinical results have been observed in human beings [26] and horses [16] using PC with 300–400 X 10^3^ PLT/μL.

Another fact that generates controversy is the presence of leukocytes in PC. Some researches consider that WBC are a contaminant of PRP and possibly deleterious for tissues when treated, especially at high concentrations. However, others believe that WBC are important regulatory cells contained in PRP and necessary for wound healing [27]. To date, there are no studies that definitively elucidate the significance of leukocytes in PRP. However, when manual methods are used for producing PC in human beings and horses, leukocyte concentrations are comparatively lower when semi-automated methods are used. Unfortunately, there is no data about WBC concentration in canine PC obtained by manual or semi-automated methods. However, it is important to note that platelet concentration of the protocol described here was not correlated with WBC concentration. This situation is different for PC derived from equine blood [24].

Both PC obtained in this study permitted concentrate two (PC-B) and three (PC-A) fold the concentration of TGF-β_1_ respect to the basal concentration of this protein in plasma. These findings suggest that plasma platelets were scarcely activated at the moment of blood extraction, since the MPV remained lower in whole blood in comparison with the same parameter in PC-A. However, although MPV value was statistically higher in PC-A in comparison with PC-B and whole blood, this platelet activation parameter remained between normal range values for canine platelets [28]. Maybe, the reason, which MPV was higher in PC-A, is related to the large number of concentrated PLT and WBC. A single centrifugation process produces cellular friction that could be most active toward the platelet fraction near to the erythrocyte package. This situation has also been observed in equine PC obtained by simple and double centrifugation tube methods [24].

Plasma TGF-β_1_ concentrations of this study were quite similar to the values described for this protein from serum of two dogs (16.5 and 19.9 ng/mL) [29]. However, other research described plasma TGF-β_1_ concentration ranging from 0.193-0.598 ng/mL. That study included 29 canine blood samples collected with EDTA by using a double centrifugation protocol [30]. Plasma or serum TGF-β_1_ concentrations described in these two last studies [29,30] could be influenced by methodological aspects such as the use or not of anticoagulant and the type of antibody used for TGF-β_1_ measurement. To note, this protein was measured in the present study with a specific canine antibody for TGF-β_1_, whereas the studies mentioned [29,30] used a human TGF-β_1_ antibody. However, this is only an assumption and further studies are necessary to validate the actual utility of human or canine ELISA kits for canine TGF-β_1_ measurement.

Plasma and both PC of this study presented higher concentrations of TGF-β_1_ in comparison with the results of the same protein from autologous conditioned plasma (ACP) and plasma obtained with ACD-A from blood of dogs [31]. In that research [31], a TGF-β_1_ mean concentration of 1.24 ± 0.59 ng/mL was obtained from ACP with PLT counts ranging from 277–293.5 X 10^3^/μL. However, the ELISA human kit used did not detect plasma TGF-β_1_ concentration. In addition, no statistical correlation was found between the number of PLT concentrated and the TGF-β_1_ concentrations [31]. This last result was different from the findings of the study described here; since strong correlations (70%) were noticed between PLT counts and TGF-β_1_ concentration. The difference between the results obtained in that study [31] and the findings of the research presented here could be related to the specificity of the antibody used for TGF-β_1_ detection and because they used no activating substances for stimulating the release of growth factors from platelets.

There is some controversy about the need of adding activating substances to induce the release of GF contained in PLT. Some researchers think that activating substances are not necessary when PC will be used as an injection for the treatment of tendon and ligament lesions or arthropaties [32]. They argue that connective tissues are rich in collagen and that this autologous protein is enough to induce platelet activation. Other researchers believe that the use of activating substances is necessary to stimulate the massive release of growth factors in the foci of the lesion and thus increasing the healing process of the affected tissue [32]. However, to date there is no scientific information to determine if activating substances should be used before PC injection.

Platelet activating substances are a necessary pre-requisite for producing platelet gel from PC. Platelet gels are used for covering large skin defects or for filling bone defects either alone or combined with other biomaterials. Platelet gels could be produced from PC activated with proteic and non-proteic substances or by combination of both. Classically, bovine thrombin (either alone or in combination with a calcium salt) has been used for platelet gel production. Thrombin induces fibrin polymerization by removing fibrinopeptides A and B from the fibrin molecule. This protein also produces platelet activation and massive release of growth factors. Some clinicians prefer not to use bovine thrombin for platelet gel formation because this substance induces cross-reacting antibodies against coagulation factors V and XI [9].

Batroxobin induces fibrin polymerization by removing only the fibrinopeptide A. This substance does not induce platelet activation or immunological reactions against coagulation factors [9]. The manufacturer recommends the use of batroxobin plus calcium gluconate for platelet gel formation. In the study presented here no differences were noticed on the release of TGF-β_1_ from both PC activated with CG or batroxobin plus CG. Macroscopically no differences were noted about the quality of the platelet gel formed or the time required for clot formation. However, one limitation of this study is that kinetics of gelation was not performed [9].

Results of this study corroborate that batroxobin induce negligible platelet activation and the addition of CG was necessary to induce the TGF-β_1_ release. This phenomenon could be explained without the need of using an experimental group of PC activated only with batroxobin, since TGF-β_1_ concentration was statistically similar for both PC, independently of the activating substance used, CG or batroxobin plus CG. This study is limited since TGF-β_1_ release only was measured at once. Further studies are necessary for knowing if the release kinetics of this growth factor is time dependent or is related to an activating substance in particular.

Collection and concentration efficiencies are two important aspects related with the capacity of and protocol or device for concentrating the most possible number of platelets and growth factors from a whole blood sample. The protocol described here for producing PC and consequently concentrating TGF-β_1_ was better than the cellular and molecular results obtained for canine ACP protocol [31] and the platelet concentration efficiency described for a semi-automated method for producing canine PRP [23].

## Conclusions

In summary, this study describes a simple and inexpensive manual tube protocol for producing two PC with different cellular and molecular characteristics. Release of TGF-β_1_ from both PC at 2 h post-activation was mainly dependent of CG. Further research is necessary to determine the quality and strength of the platelet gel formed before PC activation with CG or batroxobin (either alone or in combination with CG). A study on the kinetics of release of TGF-β_1_ and other platelet storage growth factors from PC activated with several substances during several hours should be performed.

Clinical studies are necessary to determine the actual clinical utility of each PC produced with the protocol of this study. Aspects related with the cellular or molecular composition of PC possibly will determine the specific use of each biodrug for a particular disease or tissue. The use of platelet activating substance also will determine the specific clinical use of PC in dogs.

## Methods

The ethics committee of animal research of Federal University of Minas Gerais approved this study.

### Animals

Sixteen male mongrel dogs were used, with age range between 16 to 24 months and 15 Kg of average weight, clinically healthy at time of blood collection and serologically negative for leishmaniasis and ehrlichiosis.

### Preparation of platelet concentrates

Blood collection was performed by puncturing the saphenous vein with a butterfly catheter 21 G (Shandong Weigao Group, Weihai, China). Blood was placed in 8.5 mL tubes with 1.5 mL of ACD-A (trisodium citrate 22 g/L, citric acid 8 g/L and dextrose 24.5 g/L) (Becton Dickinson and Company, New Jersey, USA). Tubes were centrifuged (SIGMA 3 K30, Osterode am Harz, Germany) at 191 g for 6 minutes. Arbitrarily, the plasma derived from blood centrifugation was divided in two equal fractions, PC-A and PC-B. PC-A (lower PC fraction) was considered as the first 50% of plasma next to the packed cell volume (PCV) and PC-B (upper PC fraction) was the 50% of remaining plasma in the tube.

### Cellular evaluation

Blood samples and both PC were analyzed for hemogram by using an automated counting device by volumetric impedance (Abacus Junior Vet, Budapest, Hungary). Each sample was analyzed by triplicate. The hematological parameters tested were hematocrit (PCV), platelet count (PLT/μL), leukocyte count (WBC/μL), absolute counts (cells/μL) and relative values (% cells) of lymphocytes (LYM), monocytes (MID), neutrophils, eosinophils and basophils (GRA), mean platelet volume (MPV fL) and platelet distribution width (PDW %).

### Activation of platelet concentrates

Samples of one mL from PC-A and PC-B were divided into aliquots of 500 μL and then activated with 50 μL of CG 10% (Ropsohn Therapeutics Ltda, Bogotá, Colombia) or batroxobin (Plateltex, Praha, Czech Republic) reconstituted with one mL of CG 10% (Ropsohn Therapeutics Ltda, Bogotá, Colombia). After activated, the samples were kept in incubation at room temperature (22°C) for two hours [24]. Fibrin clots in each PC sample were released from the tube walls and centrifuged at 1500 g for 10 minutes. Further, others plasma samples were obtained by the centrifugation protocol described above. The supernatants of activated PC and plasma samples were aliquoted and frozen at - 80°C for later determination of TGF-β_1_ concentration.

### Determination of total protein

Total protein (TP) concentration was measured by duplicate in both PC and plasma by using the biuret method (Biosystems, Barcelona, Spain) in a semiautomatic chemistry analyser (RT-1904CV, Nanjing, China). This determination was performed to know the proportion of TGF-β_1_/TP released during PC activation.

### Determination of the concentration of transforming growth factor beta 1

Concentration of TGF-β_1_ (ng/mL) in plasma and both PC were determined by ELISA sandwich, specifically developed with antibodies against canine TGF-β_1_ (Mouse/Rat/Porcine/Canine TGF-β_1_, MB100B, R&D Systems, Minneapolis, USA). This protein had a mean detection sensitivity of 4.6 pg/mL. ELISA was performed by duplicate for each sample according to the manufacturer instructions. Reading (Biochrom, Anthos 2010, Cambridge, UK) was performed at 450 nm.

### Statistically analysis

Data derived from this study presented normal distribution (Shapiro-Wilk test, P > 0.05) and analyzed variables were presented as mean and mean standard error. Comparison between groups was performed using a one-way ANOVA and *post-hoc* par-wise comparisons were performed with a Student-Newman-Keuls (SNK) test. Correlations between TGF-β_1_ concentrations and cellular data were performed using a Pearson (ρ) test. A value of P ≤ 0.01 was accepted as statistical significant for all the tests.

### Collection (concentration) efficiency

Platelet collection efficiency was determined by the formula: (PC volume x platelet count in the PC/whole blood volume x platelet count in whole blood) x 100 [33].

TGF-β_1_ concentration efficiency was determined by the formula:(concentration of TGF-β_1_ in PC (ng/mL) x volume of PC/plasmaTGF-β_1_ concentration (ng/mL) x whole blood volume) x 100 [34].

## Authors’ contributions

RFS conceived of the study, performed the laboratory tests, performed the statistical analysis and participated in the drafting of the manuscript. CMFR participated in the design and participated in the drafting of the manuscript. JUC coordinated the study, participated in the design and harmonized the drafting of the manuscript. All authors read and approved the final manuscript.
